# 4-Ports endoscopic extraperitoneal radical prostatectomy: preliminary and learning curve results

**DOI:** 10.1590/S1677-5538.IBJU.2015.0323

**Published:** 2016

**Authors:** Humberto do Nascimento Barbosa, Tiberio Moreno Siqueira, Françualdo Barreto, Leonardo Gomes Menezes, Mauro José Catunda Luna, Adriano Almeida Calado

**Affiliations:** 1 Departamento de Urologia da Universidade de Pernambuco - Recife, PE, Brasil;; 2 Departamento de Urologia, IMIP, Recife, PE, Brasil;; 3 Serviço de Urologia, Memorial São José Hospital, Recife, PE, Brasil

**Keywords:** Endoscopy, Prostatectomy, Learning, Minimally Invasive Surgical Procedures

## Abstract

**Introduction:**

There is a lack of studies in our national scenario regarding the results obtained by laparoscopic radical prostatectomy technique (LRP). Except for a few series, there are no consistent data on oncological, functional, and perioperative results on LRP held in Brazil. As for the LRP technique performed by extraperitoneal access (ELRP), when performed by a single surgeon, the results are even scarcer.

**Objective:**

To analyze the early perioperative and oncologic results obtained with the ELRP, throughout the technical evolution of a single surgeon.

**Patients and methods:**

A non-randomized retrospective study was held in a Brazilian hospital of reference. In the 5-year period, 115 patients underwent the ELRP procedure. Patients were divided into two groups, the first 57 cases (Group 1) and the following 58 cases, (Group 2). A comparative analysis between the groups of efficacy results and ELRP safety was carried out.

**Results:**

The average age of patients was 62.8 year-old and the PSA of 6.9ng/dl. The total surgery time was 135.8 minutes on average, and the urethral-bladder anastomosis was 21.9 min (23.3 min versus 20.7 min). The positive surgical margins (PSM) rate was 17.1%, showing no difference between groups (16.4% versus 17.9%; p=0.835). There was statistical difference between the groups in relation to the anastomosis time, estimated blood loss and the withdrawal time of the urinary catheter.

**Conclusion:**

The ELRP technique proved to be a safe and effective procedure in the treatment of prostate cancer, with low morbidity.

## INTRODUCTION

The first series of laparoscopic radical prostatectomy (LRP) was described by Schuessler et al (1) in 1997. Nine surgeries were performed by the transperitoneal technique (TLRP). In the year 1997, Raboy et al (2) described the extraperitoneal laparoscopic radical prostatectomy (ELRP).

The first ones to carry out the ELRP procedure in Brazil were Andreoni et al (3) in 2001. They had an exceptional higher incidence of hypercapnia with conversion, maybe due to longer operative time. No functional and oncological results were provided. The first successful nationwide series of ELRP procedures was described by Tobias-Machado et al (4) in 2004.

In the 2010 cohort study, Guillonneau et al (5) observed that the percentage of positive surgical margins (PSM) gets stabilized after the performance of 250 cases, occurring in 22% of patients. They concluded that, previous experience in retropubic radical prostatectomy (RRP) did not influence the result of the PSM, and that this rate decreases more slowly during the learning curve (LC) in LRP compared with the open technique. Thus, it suggests that the results depend directly and exclusively of the laparoscopic technique training.

In a series of 760 cases, Mirandolino et al (6) found that the percentage of PSM in LRP was similar to the studies using the open technique. The PSM average was from 11% to 26%. In five years time, there was a biochemical recurrence rate of 11%.

In a study comparing the LC from different surgeons, Siqueira et al (7) observed that the rate of complications from both ELRP and TLRP techniques were similar. On the other hand, the overall rate of PSM was lower when using the TLRP technique.

In a nationwide series, with 270 cases performed by different surgeons, Starling et al (8) observed that there was a drop from 15% to 10% in the PSM rate after a LC of 70 cases.

There is a lack of national studies with respect to the results obtained from the LRP technique. Except for the series of the cases described above, there are no consistent data on oncological, functional and perioperative results on LRP held in Brazil. Concerning the ELRP technique when performed by a single surgeon, the results are even scarcer.

The present study analyses the early perioperative and oncologic results obtained with ELRP performed by a single surgeon.

## MATERIALS AND METHODS

It was a backward-looking, non-randomized study held in a Brazilian hospital of reference. It only represents the first 115 cases (with patients operated from 2008 through 2013), who underwent the laparoscopic extraperitoneal technique, using only a four-port. It does represent the learning curve of a single surgeon in ELRP technique. All patients had PSA<8, clinical stage T1 and T2. The initial 40 cases performed by TLRP technique were excluded. The patients involved in the study were divided into two groups, the first 57 cases were called Group 1 and the 58 following cases were called Group 2.

**Figure 1:**
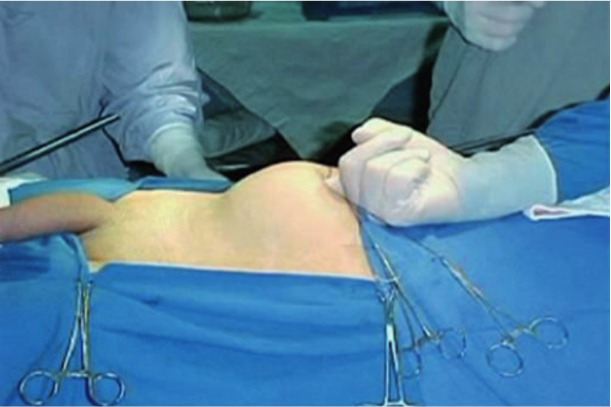
– Digital dissection of the pre-peritoneal area.

**Figure 2:**
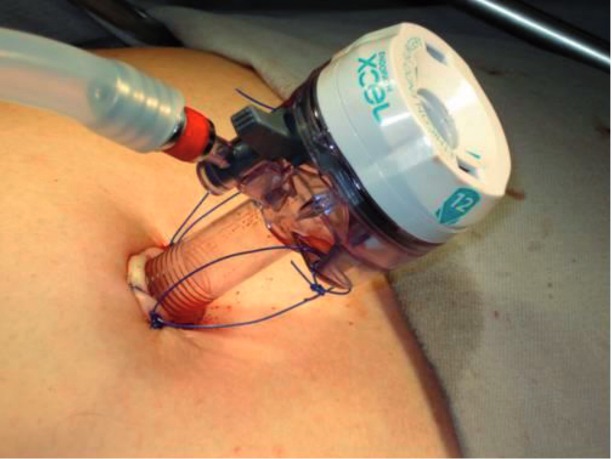
– Port closure with an umbilical point “X” on each side of the optical trocar.

**Figure 3:**
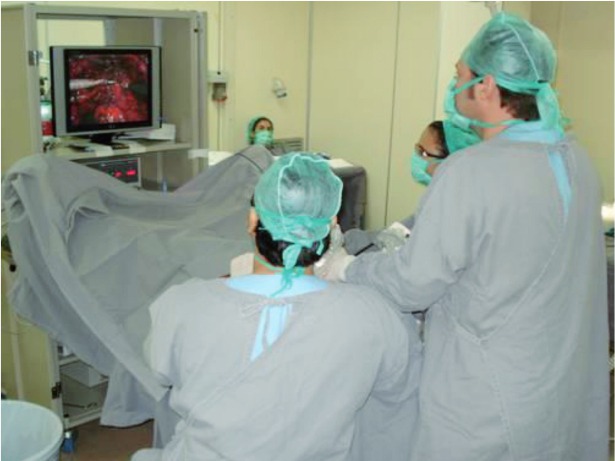
Patient and surgical team positioning.

**Figure 4:**
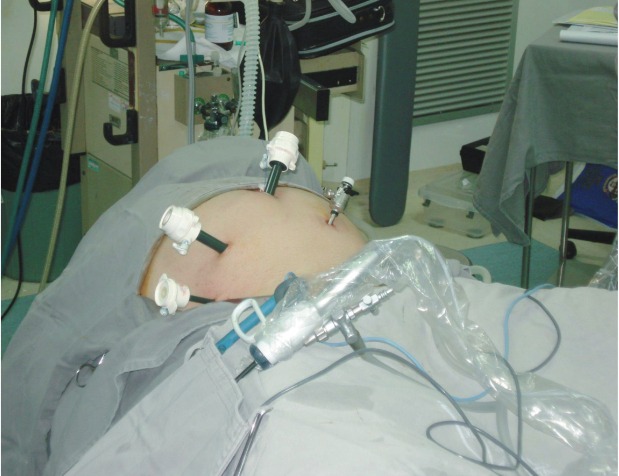
Arrangement of ports when the first assistant surgeon is left-handed.

The configurations of the ports were the same for all the cases. The camera port was done by an infraumbilical incision of about 4cm, followed by the digital dissection of the pre-peritoneal space and closed with Prolene thread (Figures 1 and 2). After Trendelenburg position (Figure-3), the surgeon’s work port was inserted into the edge of the rectus abdominis muscle at the midway between the iliac crest and the umbilicus, the left port had 5mm and the right one, 10-12mm. The 5mm port of the first assistant surgeon was inserted on the left side near the iliac crest. When the first assistant surgeon was left-handed this portal stood on the right side (Figure-4).

In the surgical technique used, the extraperitoneal area is digitally created followed by the opening of the endopelvic fascia bilaterally. The complex of the dorsal penile vein is tied with polyglactin sutures 0, and the bladder neck is opened with an ultrasonic scalpel. After the dissection of the seminal vesicles and the vas deferens, the neurovascular bundles are separated from the prostate with the use of ultrasonic energy or by placing polymer clips (Hem-O-Lok^®^), followed by the section with laparoscopic scissors, always using the preservation of nerves interfascial technique. After the removal of the prostate, the urethra is anastomosed to the bladder neck with a polyglactin 3.0 suture, making use of the Van Velthoven (9) technique.

A comparative analysis between groups of early perioperative and oncological outcomes was performed. In order to compare the groups regarding the qualitative variables, the chi-square test of Pearson or the Fisher exact test were used. In the cases where the normal distribution assumption was not rejected, the t-Student test was applied. As for the normality rejection situations, the nonparametric Mann-Whitney test was used.

## RESULTS

Table-1 shows the results of the characterization of the entire sample as well as for each Group. The groups were homogeneous, with no difference between them. The average age of patients was 62.8 years old, with the average preoperative PSA of 6.9ng/ml, and average prostate weight of 39.3g taken by the trans-rectal ultrasound. The majority of cases (80.9%) were diagnosed solely due to the increase of PSA in routine tests (clinical stage T1c) and in most cases with a Gleason 6 (50.4%) and Gleason 7 (40%), respectively. Table-2 shows the results of oncological data according to D’Amico classification (10).

**Table 1:** – Characterization of the entire sample.

	Total (n=115)	Group 1 (n = 57)	Group 2 (n = 58)	p
**Age (years):** average (±SD)	62.8 (±7.3)	62.8 (±8.0)	62.7 (±6.5)	0.941_t_
**ICM (kg/m** ^**2**^ **):** average (±SD)	27.2 (±3.4)	21.1 (±3.3)	27.3 (±3.6)	0.827_t_
**PSA (ng/ml):** average (±SD)	6.9 (±4.4)	7.1 (±4.5)	6.6 (±4.4)	0.510_t_
**weight (g):** average (±SD)	39.3 (±13.0)	40.8 (±14.9)	37.8 (±10.9)	0.070_t_
**Nodule:** n (%)	22 (19.1%)	12 (21.1%)	10 (17.2%)	0.603
**Clinic Stage:** n (%)				0.775_F_
T1b	1 (0.9%)	1 (1.8%)	-	
T1c	93 (80.9%)	45 (78.9%)	48 (82.8%)	
T2a	13 (11.3%)	6 (10.5%)	7 (12.1%)	
T2b	8 (7.0%)	5 (8.8%)	3 (5.3%)	
**Gleason Score:** n (%)				0.173_F_
4 (2+2)	1 (0.9%)	1 (1.8%)	-	
5 (2+3)	1 (0.9%)	1 (1.8%)	-	
5 (3+2)	3 (2.6%)	1 (1.8%)	2 (3.4%)	
6 (3+3)	58 (50.4%)	23 (40.4%)	35 (60.3%)	
7 (3+4)	34 (29.6%)	18 (31.6%)	16 (27.6%)	
7 (4+3)	12 (10.4%)	9 (15.8%)	3 (5.2%)	
8 (3+5)	1 (0.9%)	1 (1.8%)	-	
8 (4+4)	5 (4.3%)	3 (5.3%)	2 (3.4%)	

**SD** = standard deviation; **t** = T-Student test; **F** = Fisher’s exact test; **ICM** = index of corporal mass

**Table 2:** – D’Amico risk stratification groups for prostate cancer.

Risk Escore	Total (n=115)	Group 1 (n = 57)	Group 2 (n = 58)	p
Low Risk	48 (41.7%)	19 (33.3%)	29 (50.9%)	0.171
Intermediate Risk	56 (48.7%)	31 (54.4%)	25 (43.1%)	
High Risk	11 (9.6%)	7 (12.3%)	4 (6.9%)	

Table-3 shows the results of the intraoperative variables of the evaluated technique. The total surgical time was on average 135.8 min. with the UV anastomosis time average of 21.9 min. In one case, there was conversion to transperitoneal technique. There was no statistical difference between the groups when comparing the number of conversions to open surgery, as well as when compared surgical complications.

**Table 3:** – Intraoperative and post-operative variables.

	Total (n=115)	Group 1 (n = 57)	Group 2 (n = 58)	p
**Anastomosis Time (min):** Average (±SD)	21.9 (±6.2)	23.3 (±6.9)	20.7 (±5.2)	0.027_t_^(*)^
**Blood Transfusion: n (%)**	4 (±3.5%)	2 (±3.5%)	2 (±3.4%)	> 0.999_F_
**Surgical Time (min):** Average (±SD)	135.8(±34.3)	139.5 (±32.8)	132.3 (±35.7)	0.269_t_
**Estimated Bleeding Rates (ml):** Average (±SD)	178.4(±80.9)	200.7 (±89.5)	156.4 (±65.0)	0.003_M_^(*)^
**Conversion Number:** n (%)	5 (4.3%)	3 (5.3%)	2 (3.4%)	0.679_F_
**Time in the Hospital** (days) Average (±SD)	2.2 (±0.5)	2.3 (±0.7)	2.0 (±0.2)	0.022_t_^(*)^
**Urinary Catheter Time** (days): Average (±SD)	9.7 (±2.6)	10.6 (±2.9)	8.8 (±1.8)	<0.001_M_^(*)^

**T =** T-Student test; **F** = Fisher’s exact test; **M** = Mann-Whitney test

From the results (Table-3), we can see that there was a significant reduction in the time of anastomosis and bleeding estimated between groups in favor of Group 2. On the other hand, there was no statistical difference regarding the need for blood transfusion. We can see that there was a significant reduction in the hospital stay and the urinary catheter removal time between the two groups, also in favor of Group 2.

Among the complications, there was no statistical difference between the groups (p=0.92). Table-4 shows all the complications that happened in this study according to Clavien-Dindo classification system (11).

**Table 4:** – Complications according to Clavien-Dindo classification system.

Complication	Total (n=115)	Group 1 (n = 57)	Group 2 (n = 58)	p
**No complications**	83 (72.2%)	40 (70.2%)	43 (74.1%)	0.924_F_
**Urinary infection** (grade II)	14 (12.2%)	6 (10.5%)	8 (13.8%)	
**Seroma** (grade IIIa)	1 (0.9%)	1 (1.8%)	0 (0%)	
**Urinary extravasation** (grade I)	2 (1.7%)	2 (3.5%)	0 (0%)	
**Anastomosis rupture** (grade IIIa)	1 (0.9%)	0 (0%)	1 (1.7%)	
**Hematuria** (grade I)	1 (0.9%)	0 (0%)	1 (1.7%)	
**Urinary retencion**	1 (0.9%)	1 (1.8%)	0 (0%)	
**Bleeding from the drain** (grade I)	1 (0.9%)	1 (1.8%)	0 (0%)	
**Urethral stricture** (grade IIIa)	2 (1.7%)	1 (1.8%)	1 (1.7%)	
**Bladder neck stenosis** (grade IIIa)	2 (1.7%)	1 (1.8%)	1 (1.7%)	
**Blood transfusion** (grade II)	4 (3.5%)	2 (3.5%)	2 (3.4%)	
**Rectal injury** (grade IIIb)	2 (1.7%)	1 (1.8%)	1 (1.7%)	
**Epigastric artery injury** (grade IIIb)	1 (0.9%)	1 (1.8%)	0 (0%)	

Table-5 analyzes the variables related to the postoperative pathologic evaluation. No statistical difference was observed between the groups. In relation to the Gleason scores, the most prevalent was (3+4) in 45.9% of the cases. The perineural invasion could be observed in 24.5% of cases, and the invasion of the prostate capsule in 13.5%. The positive surgical margin variable (PSM) was 17.1%, with no difference between groups (16.4% versus 17.9%; p=0.835).

**Table 5:** – Postoperative pathologic evaluation.

Variables	Total (n = 111)*	Group 1 (n = 55)	Group 2 (n = 56)	P
**Gleason Escore:** n (%)				0.497_F_
5 (2+3)	1 (0.9%)	1 (1.8%)	-	
5 (3+2)	2 (1.8%)	-	2 (3.6%)	
6 (3+3)	34 (30.6%)	18 (32.7%)	16 (28.6%)	
7 (3+4)	51 (45.9%)	22 (40.0%)	29 (51.8%)	
7 (4+3)	15 (13.5%)	9 (16.4%)	6 (10.7%)	
8 (4+4)	4 (3.6%)	2 (3.6%)	2 (3.6%)	
9 (4+5)	4 (3.6%)	3 (5.5%)	1 (1.8%)	
**Perineural Invasion:** n (%)	27 (24.5%)	14 (26.4%)	13 (22.8%)	0.660
**Prostate Capsule Invasion:** n (%)	15 (13.5%)	5 (9.1%)	10 (17.9%)	0.177
**Positive Surgical Margin:** n (%)	19 (17.1%)	8 (16.4%)	11 (17.9%)	0.835
**Pathological Stage:** n (%)				0.177

**F** = Fisher’s exact test

*= 02 cases with benign prostatic hyperplasia result were excluded and 02 cases in which the operative specimen were lost.

In terms of disease staging, 86.5% of cases were classified as pT2, and 13.5% were classified as pT3. There was no difference between groups when analyzing the variable pathologic stage (p=0.17).

Table-6 shows that there was significant change in the Gleason score in the pre and postoperative period. There was an increase of 38.6% of the Gleason 6 (3+3) to the Gleason 7 (3+4), and of 14% for Gleason 7 (4+3), showing a understaging on the preoperative Gleason score, when analyzing the surgical specimens.

**Table 6:** Postoperative Gleason.

Postoperative Gleason																			
**Preoperative Gleason**			**2+3**	**3+2**	**3+3**	**3+4**	**4+3**	**4+4**	**4+5**	**Total**
	2+2	N	0	0	1	0	0	0	0	1
		%	0.0%	0.0%	100%	0.0%	0.0%	0.0%	0.0%	100%
	2+3	N	1	0	0	0	0	0	0	1
		%	100%	0.0%	0.0%	0.0%	0.0%	0.0%	0.0%	100%
	3+2	N	0	0	0	2	0	0	1	3
		%	0.0%	0.0%	0.0%	66.7%	0.0%	0.0%	33.3%	100%
	3+3	N	0	1	26	22	8	0	0	57
		%	0.0%	1.8%	45.0%	38.6%	14%	0.0%	0.0%	100%
	3+4	N	0	1	4	21	2	1	2	31
		%	0.0%	3.2%	12.9%	67.7%	6.5%	3.2%	6.5%	100%
	3+5	N	0	0	0	0	0	1	0	1
		%	0.0%	0.0%	0.0%	0.0%	0.0%	100%	0.0%	100%
	4+3	N	0	0	2	4	4	1	1	12
		%	0.0%	0.0%	16.7%	33.3%	33.3%	8.3%	8.3%	100%
	4+4	N	0	0	1	2	1	1	0	5
		%	0.0%	0.0%	20.0%	40.0%	20.0%	20.0%	0.0%	100%
Total		N	1	2	34	51	15	4	4	111
		**%**	**0.9%**	**1.8%**	**30.6%**	**45.9%**	**13.5%**	**3.6%**	**3.6%**	**100%**

**p-value** = 0.001

Table-7 shows the incidence of PSM regarding the pathological stage. We observed that 8.3% of the cases with pT2 stage, and 73.4% of the cases with pT3 stage showed PSM (p<0.001).

**Table 7:** – Incidence of PSM regarding the pathological stage.

Surgical Margins	Pathological Stage			P
**(Group 1)**	**pT2**	**pT3**	**Total**	
**Negative**	88 (91.7%)	04 (26.7%)	92 (82.9%)	0.001_F_
**Positive**	08 (8.3%)	11 (73.4%)	19 (17.1%)	
**Total**	96 (100%)	15 (100%)	111 (100%)	

**F** = Fisher’s exact test

There was no difference in the incidence of PSM for Groups 1 and 2, when the PSM rate was analyzed by separate groups (Table-8). Furthermore, in Group 2 there was a statistical trend of positive margin with pT3 stage (Table-9).

**Table 8:** Incidence of PSM for groups 1 and 2.

Surgical Margins	Pathological Stage			P
**(Group 1)**	**pT2**	**pT3**	**Total**	
	
Negative	44 (95.7%)	02 (4.3%)	46 (100%)	0.027_F_
Positive	06 (66.7%)	03 (33.3%)	09 (100%)	

**Total**	**50 (90.9%)**	**05 (9.1%)**	**55 (100%)**	

(Group 2)	pT2	pT3	Total	
	
Negative	44 (95.7%)	02 (4.3%)	46 (100%)	<0.001_F_
Positive	02 (20.0%)	08 (80.0%)	10 (100%)	

**Total**	**46 (82.1%)**	**10 (17.9%)**	**56 (100%)**	

**F** = Fisher’s exact test

**Table 9:** - Comparison of groups in relation to the final stage (only for patients with positive margins)

		Final stage		Total
		pT2 n = 8	pT3 n = 11	N = 19
Group	1	6 (66.7%)	3 (33.3%)	9 (100%)
	2	2 (20.0%)	8 (80.0%)	10 (100%)

**p-value** = 0.070 (Fisher’s exact test)

Through the result above, a statistical trend can be observed, (p<0.10) difference between the groups (80% vs. 33%).

## DISCUSSION

In all three technical procedures (open, laparoscopic or robotic), there is a specific goal which is the healing treatment of localized prostate cancer (12). Some authors argue that in the TLRP the initial dissection of the seminal vesicles and vas deferens facilitates the dissection step and the preservation of the neurovascular bundle. On top of that it is easier as it promotes more physical space and light, and it also allows greater visibility of the anatomical structures leading to less tension in the anastomosis (13).

On the other hand, the ELRP brings a similar procedure to the conventional retropubic one, while maintaining the integrity of the peritoneum, allowing for less possibility of intra-abdominal complications (2). Therefore, this access is defined as the safest one as it does not violate the peritoneal cavity (14-16).

The robotic assisted laparoscopic radical prostatectomy extraperitoneal access (RALRP-EX) is just used in a few institutions, as it holds a long and difficult LC. This technique provides very limited space for the robotic movements, and difficult to make lymphadenectomy. It is recommended to be just started by surgeons with extensive experience in transperitoneal robotic assisted laparoscopic radical prostatectomy (RALRP-TP) (14).

According to Mitre et al, high costs, lack of accessibility to training and reduced budgets are the biggest problems for the spread of robot technology in low-volume centers, especially in developing countries (17).

The best parameter to evaluate the oncologic efficacy is disease-free survival, but with the impossibility to assess this parameter due to short segments, the recurrent biochemical rate is the most appropriate way, and is directly associated with the PSM rate (18).

According to Guillonneau et al (5), the percentage of PSM only stabilized after 250 cases performed in LRP, with an incidence of 22%. It was suggested that these results are due to the training in laparoscopy (19).

As oppose to the above study, our series with only 115 cases had the overall rate of PSM of 17.1%, with no significant difference between groups. Thus, the continuous learning in the extraperitoneal technique did not influence the oncological results obtained from surgical specimens. We also observed that most cases of PSM occurred in the pathological stage pT3 (73.4%). If we just analyze the cases of pT2 stage, we can see a very low PSM rate (8.3%), below the average of published data.

Similarly to our results, Mirandolino et al (6) reported that the PSM average obtained in cases under LRP was from 11%-26%.

In general, it is observed that during the LC, the perioperative results are lower than the ones observed with large laparoscopic or RRP series. Such results begin to improve after the learning period, which happens around 10-80 cases (20, 21). However, the sufficient number of surgeries to bridge this period may be higher when the oncological and functional results are also evaluated. In the present study, the initial 40 cases performed by transperitoneal access, were excluded to obtain two comparable and homogeneous groups, in which all the surgeries were performed by the same surgical technique, via the extraperitoneal access and by the same surgeon.

According to Leroy et al (22) the fellowship training in robotics, considerably improves the LC in the RALRP and that in the first 30 surgeries performed by the group in which there was training, the PSM rate was significantly lower, 15% versus 34%. At the same time, Kown et al (23) demonstrated that the LC in robotics is extremely short, with only 25 procedures. On the other hand, Peters et al (24) reported that the PSM rate improved significantly after performing 800 robotic surgeries. However, their LC only finished with the average of 1600 procedures, showing a PSM rate lower than 10%.

Novarra et al (25) reported the oncological aspects related to RALRP technique in meta-analysis. In that article, the general average of PSM in RALRP was of 15%, and when stratified for the pT2 stage it was 9%. Despite the quality of the meta-analysis described above, Picozzi et al (26) found selectivity and the heading of some cases for treatment with robotic technology.

Alongside with the critics of Picozzi et al (26), an article entitled “PRLRA–fake innovation or the real deal?”, recently written by Albertsen (27), questions the indiscriminate use of robotics technology in detriment to the benefit on patients. Emanuel (28) strongly criticizes the robotic technique, and refers to RALRP as a “pseudo-innovation” and also, as “a technology that dramatically increases costs without providing increase in the health of patients.”

In our series, when we analyze the surgical performance data from both groups, we can verify that the average time to perform anastomosis, the time for the urinary catheter removal and estimated blood loss were much lower in Group 2 than in Group 1. These data suggest that there was a technical improvement during the course of time, reflecting the learning process of the extraperitoneal technique. Even so, we found that the estimated bleeding rates for both groups were at the lower limit of the rates found in the literature, ranging from 201.5 to 1323mL in the ELRP and 69-534ml in the RALRP (29).

In our series there was no conversion to open surgery due to bleeding. All conversions occurred due to technical difficulties, mainly because of obesity and retropubic adhesions, the latter being probably caused by the inflammatory process associated with the prostate biopsy. The creation of the physical space in the extraperitoneal access can be obtained to some technical difficulties in some cases. In this series specifically, there was one case in one patient during the creation of the retroperitoneal space, a tiny perforation in the peritoneal envelope. Therefore, the conversion to TLRP was necessary.

There were no cases of hypercarbia because the operative time was not too long (average of 135.8 min.), probably due to the previous experience in TLRP procedures. Therefore, the learning curve is more difficult than in the TRLP technique, but gradually overcome with the previous experience in laparoscopy.

I believe that ELRP could be taught at residency or special programs for the urological community in Brazil.

Due to the retrospective nature of this study, it was not possible to properly assess functional aspects such as sexual potency and urinary incontinence.

In our study, there were rectal lesions in two cases, which were promptly corrected intraoperatively.

Although the anastomosis was done with continuous suture, in two cases there were prolonged urinary extravasation through the drain. These complications were treated only with prolonged bladder catheterization (average length of 14 days). In only one case, after the removal of the urinary catheter, the patient had urinary retention due to the localized edema in anastomosis.

Late complications presented in our study may be inherent to any surgical technique, by either the open or laparoscopic technique.

## CONCLUSION

It was observed during learning curve a significant reduction in the average time to perform the urethral-bladder anastomosis, the estimated blood loss and the removal time of the urinary catheter, seen in Group 2, that suggest that there was an improvement of the surgical technique with time. These data only reflect the surgeon’s learning process while using the ELRP.

There was no difference in early oncological results during the technical evolution, when analyzing the ELRP technique.
